# Morphological characteristics of mule conceptuses during early development

**DOI:** 10.21451/1984-3143-AR2017-0035

**Published:** 2018-12-05

**Authors:** Nathia Nathaly Rigoglio, Gustavo de Sá Schiavo Matias, Maria Angelica Miglino, Andrea Maria Mess, Julio Cesar Ferraz Jacob, Lawrence Charles Smith

**Affiliations:** 1 Department of Surgery, School of Veterinary Medicine and Animal Science, University of Sao Paulo, Brazil.; 2 Department of Reproduction and Animal Evaluation, Federal Rural University of Rio de Janeiro, Seropedica, Rio de Janeiro, Brazil.; 3 Department of Veterinary Biomedicine, Centre de recherche en reproduction et fertilité, Faculty of Veterinary Medicine, University of Montreal, QC J2S 2M2, Saint-Hyacinthe, QC, Canada.

**Keywords:** embryology, development, mule

## Abstract

Hybrids between species are often infertile and extremely rare among mammals. Mules, i.e. crossing between the horse and the donkey, on the other hand are very common in agricultural and leisure practices due to their enhanced post-natal physical characteristics that is believed to occur for outbreeding or hybrid vigor. Since no reports are availableon the effects of hybrid vigor during early development, this study focused on characterizing the intrauterine development of mule conceptuses during critical embryo-to-fetus transition period. Nine embryos and fetuses of early gestation, obtained after artificial insemination and transcervical flushing, were evaluated by means of gross anatomy and histology and compared to data available for the equine. We found that some events, such as C-shape turning, apearence of branchial archs, limb and tail buds, formation of primary and secondary brain vesicles, heart compartmentalization, and development of somites, occurred slightly earlier in the mule. Nonetheless, no major differences were observed in other developmental features, suggesting similarities between the mule and the horse development. In conclusion, these data suggest that the effect of hybrid vigor is present during intrauterine development in the mule, at least with regard to its maternal parent.

## Introduction

Hybrids as crossbreeds of strains, varieties, or species occur in both plants and animals. The effect of hybridization on an organism is variable; in some instances the offspring is similar to one parent or it may be intermediate between the parental traits. In addition, hybrid offspring may have characteristics that are regarded to be superior or inferior in comparison to the traits of one or both parents. Offspring superiority is regarded as outbreeding enhancements, heterosis or hybrid vigor. Briefly, hybrid vigor is a fundamental biological phenomenon characterized by superior performance of the hybrid offspring as heterozygote between the two lines over its parents that are representing the homozygote conditions. It may increase the biomass, stature, growth rate, fertility and/or fitness in a population ( Birchler et al., 2003 , 2010 ; Hochholdinger and Hoecker, 2007 ; Chen, 2010a ). Several investigations of the effects of heterosis have been performed in the rabbit, pig and cattle ( Long, 1980 ; Blasco et al., 1993; Galvin et al., 1993 ; Newman et al., 1993 ). In addition, studies on hybrids allows for investigations onthe specific role of genes in developmental attributes ( Goodwin, 2007 ). For instance, hybridization studies have revealed how epigenetic variation ( Jirtle and Skinner, 2007 ; Simmons, 2008 ; Skinner et al., 2010 ; Guerrero and Skinner, 2012; Groszmann et al., 2013 ) contributes to a better understanding of the molecular mechanisms of complex traits associated with hybrid vigor ( Cubas et al., 1999 ; Manning et al., 2006 ; Shindo et al., 2006 ; Ni et al., 2009 ; He et al., 2010 ). In domestic animal and plants, hybrid vigor seems to minimize inbreeding and to enhance breeding outcome ( Gray, 1972 ; Anderson, 1988 ).

Hybrids between true species are rare, especially among mammals. Nonetheless, the mixing of species has always fascinated humans and inspired imaginations of creatures such as sphinx and centaurs in mythology. However, due to natural barriers and isolating mechanisms, interspecies mating is successful only in a few closely related mammalian species ( McGovern, 1976 ). Examples that are able to crossbreed are known for felids, ursids and bovides as well as several species of equides. Hybrids between the horse *Equus caballus* and the donkey *Equus asinus* are common, resulting in the mule as a hybrid of a female horse and a male donkey and the hinny as the reciprocal crossbreed ( Allen and Short, 1997 ). They are usually infertile, mainly because of chromosome inconsistencies, i.e. the horse possesses 64, the donkey 62 and the hybrids 63 chromosomes that generally inhibit proper pairing during meiosis ( Camillo et al., 2003 ). Besides this, mules are commonly used in agriculture and other purposes. In particular, mules often demonstrate hybrid vigour in physical characteristics ( Travis, 1990 ). They inherit a dense musculature from their donkey farther and tend to be stronger for carrying more weight and have better endurance than a horse but are associated with the steadfast temperament and surefootedness of their donkey mother. They are usually tall, relatively slow, but without severe leg problems ( Travis, 1990 ). The mules' performance in cognition tests is significantly better than that of the parent species ( Proops et al., 2009 ). Appart from a study based on ultrasonography ( Paolucci et al., 2012 ), little is known about the morphological effects of hybrid vigor during the critical phase of intrauterine development. Therefore, our objective here was to obtain accurate information of the gross anatomy and histology of mule conceptuses and compare these to developmental patterns observed in previous equines ( Franciolli et al., 2011 ; Rodrigues et al., 2014 ). So far, no comparable data are available on donkey early ontogeny.

## Material and Methods

### Animals

All procedures using live animals were approved by an animal ethical committee from Scholl of Veterinary Medicine and Animal Science at University of Sao Paulo (CEUA/FMVZ-USP) (protocol n°2573/2012). The mule conceptuses were collected in the Faculty of Animal Science and Food Engineering, University of São Paulo (FZEA-USP) and the Universidade Federal Rural do Rio de Janeiro (UFRRJ - Seropédica). Using 1 to 2 liters of Ringer's lactate solution at 37°C (Camillo et al., 2010), eight embryos and fetuses were collected by transcervical uterine flushing from mares at days 17 to 63 after insemination ( Tab. 1 ). Samples were fixed in 4% paraformaldehyde and crown-rump length measurements of the conceptuses were obtained after the dissection of the extra-embryonic membranes. The extra-embryonic membranes of mule conceptuses at 33, 40, 51 and 63 days of gestation were analyzed macroscopically, as well as the external characteristics of embryos at 17, 25 and 33 days of gestation and fetuses at 45 and 63 days of gestation. Macroscopic appearance of the brain, lung, kidney and fetal gonads was obtained from the fetus at 63 days of gestation using a stereomicroscope (Stemi DV4, Zeiss, USA). Results were photographed with a Nikon Coolpix P510 digital camera.

**Table 1:** Crown-rump measurements according to the gestational age of embryos and fetuses. The crowm rump of these embryos/fetuses increases as they develop.

AGE (days)	Crown-rump (cm)
17	0.3
25	1.7
33	2.5
35	2.8
40	3.3
45	4.2
51	5.3
63	7.8

Light Microscopy

The brain of conceptuses with 35, 40, 45 and 63 days of gestation, as well as the lung, heart, kidney and gonads from conceptuses with 40 and 63 days of gestation were analyzed. The samples were fixed in 4% paraformaldehyde, washed in distilled water followed by dehydration in a series of ethanol solutions at increasing concentrations (70-100%) for 30 minutes each. The sections were diaphanized in xylene for 1h and embedded in paraffin (HistosecV-MERCK, Sao Paulo, Brazil). The paraffin blocks were sectioned at 5µm in an automatic microtome (Leica, RM2165, Nussloch, Germany), mounted on histological slides, and incubated at 60°C. The sections were then deparaffinized and stained with hematoxylin and eosin (H&E).

## Results

### Extra-embryonic membranes

**Figure 1:**
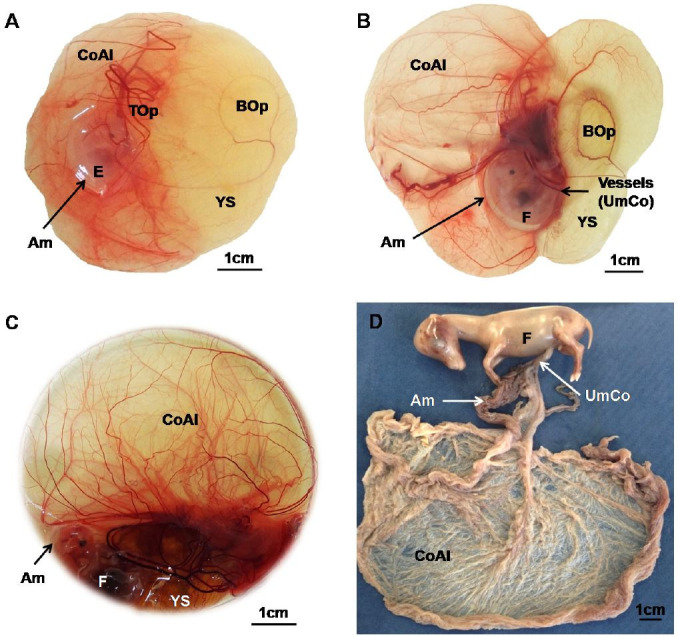
Fetal membranes of mule conceptuses at (A) 33 days, (B), 40 days, (C), 51 days, and (D) 63 days of gestation. Abbreviations: Chorioallantoic membrane (CoAl), yolk sac (YS), bilaminar omphalopleura (BOp), trilaminar omphalopleura (Top), amnion (Am), umbilical cord (UmCo).

At 17 days of gestation, the conceptus was oval in shape, loosely attached to the maternal system and had a large yolk sac. At 33 days, the large yolk sac had an extended bilaminar omphalopleura, but also possessed blood vessels forming a trilaminar omphalopleura. The chorioallantoic membrane was small, but well vascularized ( Fig. 1A ). At day 40, the yolk sac was smaller with bi- and trilaminar omphalopleura. The chorioallantoic membrane was relatively expanded ( Fig. 1B ). In the fetus of 51 days, the yolk sac was even more regressed ( Fig. 1C ). At 63 days, a yolk sac lumen was absent and the tissue was found only around the umbilical cord ( Fig. 1D ). The chorioallantoic membrane was continuously expanded ( Fig. 1C,D ). The amnion surrounded the fetus and enlarged continuously during gestation ( Fig. 1A-C ).

### External features of the conceptuses

**Figure 2:**
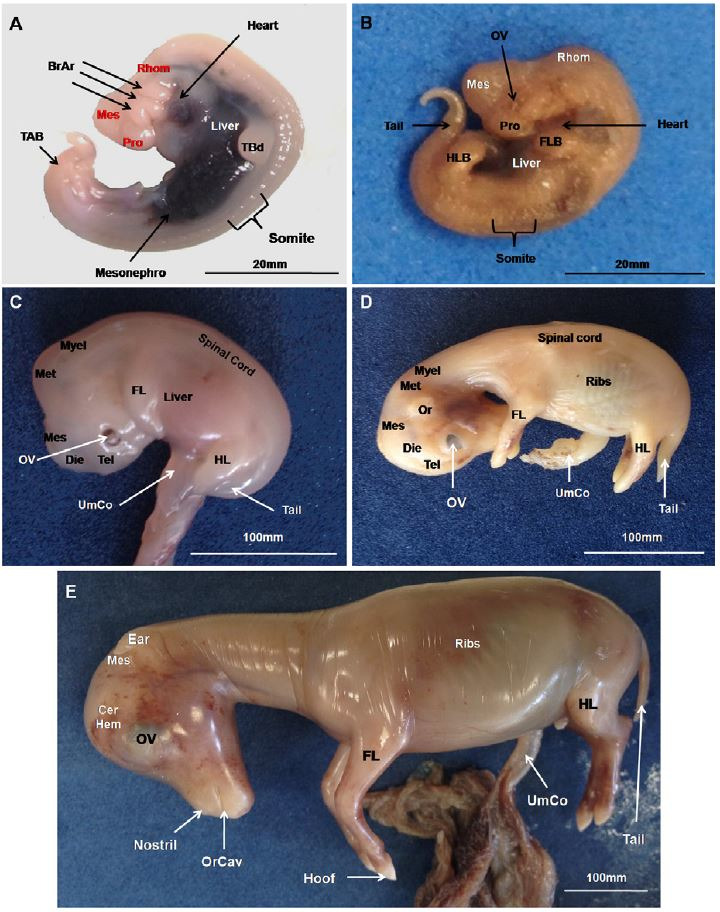
Macroscopic evaluations of mule conceptuses at (A) 17, (B) 25, (C) 33, (D) 45, and (E) 63 days of gestation. Abbreviations: Cerebral Hemispheres (CerHem), Prosencephalon (Pos), Mesencephalon (Mes), Rhombencephalon (Rhom), Telencephalo (Tel), Dyencephalon (Dye), Metencephalon (Met), Myelencephalon (Mye), Optical vesicles (OV), Forelimb buds (FLB), Hindlimb buds (HLB), tail bud (TAB), forelimbs (FL), hindlimbs (HL), umbilical cord (UmCo), oral cavity (OrCa).

Until day 33, the skin was translucent and then became opaque ( Fig. 2A-C ). Optic vesicles were apparent at day 25 ( Fig. 2B ) and became pigmented from day 33 onwards ( Fig. 2C-E ). Forelimb and tail buds were present early in development whereas the hind limb buds were first observed only at day 25 ( Fig. 2A and B ). From day 33 onwards a forelimb and hind limb could be clearly identified, and the tail at day 45 ( Fig. 2B-D ). Somites were present until day 25 ( Fig. 2A-C ). At the 45^th^ day, external ears were present and neck and nostrils could be observed ( Fig. 2D ). On day 63, lips, eyelids, hooves and genital tubercles occurred ( Fig. 2E ).

### Neural system

**Figure 3:**
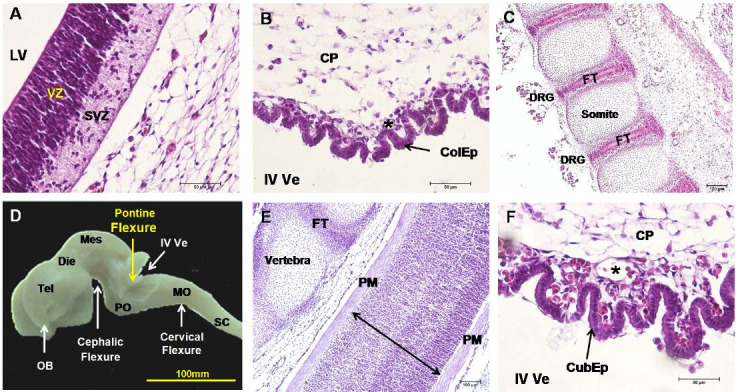
Nervous systemof mule conceptuses at 35 days (A-C), 40 days (D,E) and 45 days (F). (A) Lateral ventricle (LV) with ventricular (VZ) and subventricular zone (SVZ). (B) Choroid plexus (CP) of the fourth ventricle (IV Ve) with columnar epithelium (ColEp) and vessels (asterisks). (C) Somites with fibrous tissue (FB) and dorsal root ganglions (DRG). (D) Spinal cord surrounded by primitive meninges (PM). (E) Choroid plexus with more cuboidal epithelium (CubEp). (F) Three flexures were present.

**Figure 4:**
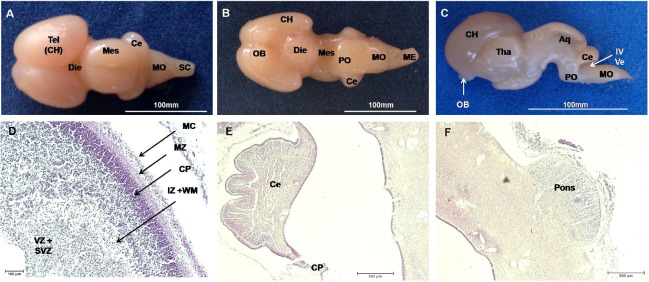
Nervous system of a mule conceptusat 63 days. (A-C) Macroscopy in dorsal, ventral and medial view showed differentiation of main brain areas. Telencephalon (Tel) with olfactory bulb (OB), Diencephalon (Die), Mesenchephalon (Mes) with aqueduct (Aq), thalamus (Tha), Metencephalon (Met),cerebellum (Ce), myelencephalon (Myel), medulla oblongata (MO), spinal cord (SC), pons (PO), and fourth ventricle (IV Ve). (D) Wall of the lateral ventricle (LV) with motor cortex (MC), marginal zone (MZ), cortical plate (CP), intermediate zone (IZ) with white matter (WM), the zone (SVZ) and ventricular zone (ZV). (E) The cerebellum with primary fissures. (F) Pons was developed.

Primary brain vesicles (forebrain, midbrain, and hindbrain) were present at day 17 ( Fig. 2A ), whereas secondary brain vesicles and the spinal cord became evident between the 35^ th^ to 45^th^ days ( Fig. 2B-D ). At 35days, ventricular and subventricular zones differentiated in the lateral ventricles ( Fig. 3A ). The choroid plexus of the fourth ventricle consisted of a single layer of columnar epithelial cells ( Fig. 3B ). Vertebrae with fibrocartilaginous tissue in between were present around the spinal cord and its dorsal ganglions ( Fig. 3C ). In the fetus of day 40, neuro-epithelium surrounded the spinal cord and the epithelium of the choroid plexus became more cuboidal, well supplied by vessels ( Fig. 3D, E ). At 45 days ( Fig. 3F ), the forebrain consisted of telencephalon with expanded cerebrum and cerebral hemispheres and diencephalon. Three flexures were found: the cephalic flexure between fore- and midbrain, the cervical flexure ventrally as well as the pontine flexure between hindbrain and myelencephalon ( Fig. 3F ). In the fetus of 63 days, lissencephalic cerebral hemispheres occurred had a prominent olfactory bulb. The thalamus occurred in the diencephalon, a mesencephalic aqueduct reached the fourth ventricle and the cerebellum occurred dorsal to bridge and the conoid medulla oblongata ( Fig. 4A-C ). The walls of the ventricle were complexly differentiated (see Fig. 4D ). The cerebellum had primary fissures and the pons was developed ( Fig. 4B,E,F ).

### Organs of thoracic and abdominal cavities

**Figure 5:**
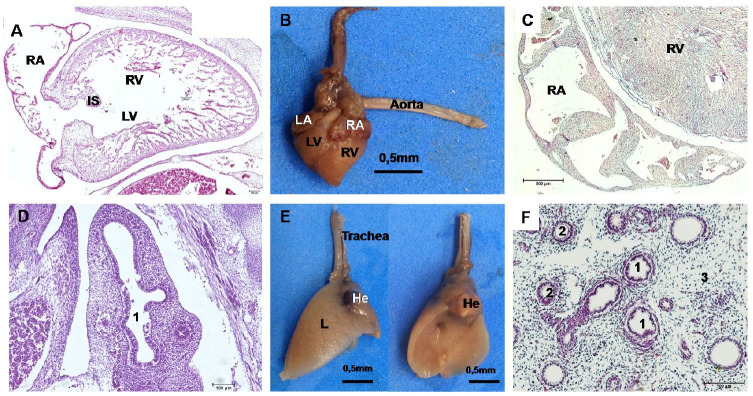
Heart and lung development in mule conceptuses. (A) Heart at 40 days with right atrium (RA), left atrium (LA), right ventricle (RV), left ventricle (LV) and intermediate septum (IS). (B) Heart in situ at day 63. (C) Atrium with valves and myocardium at day 63. (D) Lung (L) at day 40 with primary bronchi (1). (E) Lung in situ at 63 days. (F) Lung at day 63 also with secondary bronchi (2) and lung parenchyma (3).

Liver and heart were most prominent until day 33 ( Fig. 2A-C ). From day 40 onwards, the heart was in its mature position in the chest cavity along with the pericardium. It had the typical format of right and left atria and ventricles thatbegan to be separated by an intermediary septum ( Fig. 5A,B ). At 63 days, the atria had developed valves and a well-structured myocardium was present ventricles ( Fig. 5C,D ). The lung possessed the bifurcation into the primary bronchi occurred at day 40 ( Fig. 5E ) and at day 63 the lung was lobulated and had both primary and second bronchi ( Fig. 5F ).

**Figure 6:**
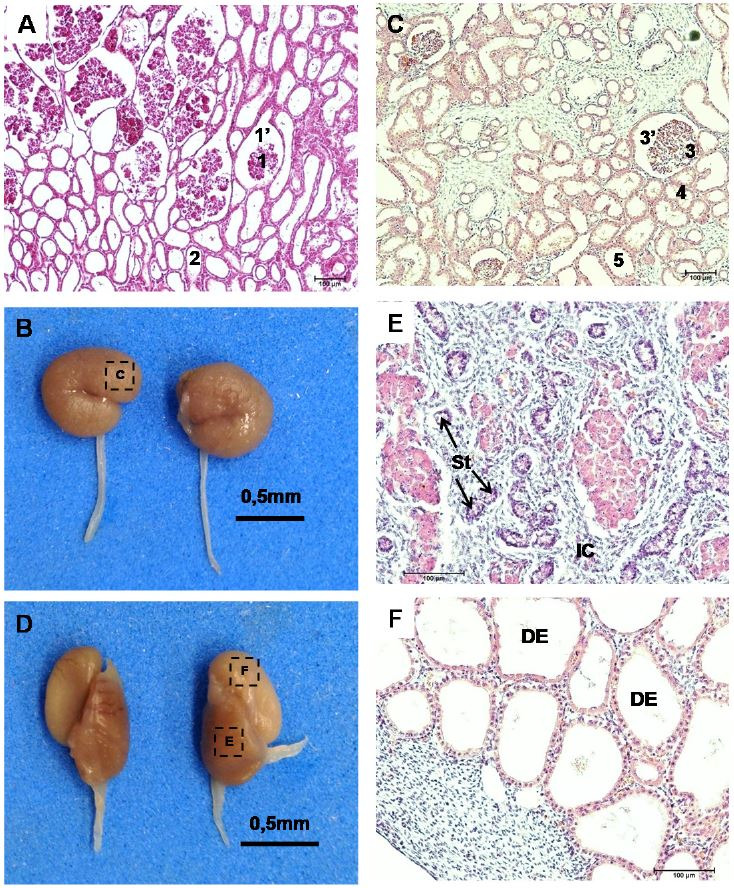
Renal and testicular development in mule conceptuses. (A) Mesonephros at 40 days of gestation with glomeruli (1) surrounded by renal capsule (1’) and mesonephric tubules (2). (B-F) Day 63. (B) Macroscopy of kidney. (C) Histology of kidney with glomerulus (3), capsule (3’), proximal convulated tubule (4), and distal convulated tubule (5). (D) Testicles and epididymis. (E) Interstitial cells (IC) and seminiferous tubules (ST) inside the testis. (F) Ductus epididymis (DE).

At day 40, some glomerulus occurred in the renal capsule, surrounded by the mesonephric tubules that had cubic epithelial cells ( Fig. 6A ). At 63 days, the metanephros had the shape of the kidney with glomeruli, capsule, proximal and distal convulated tubules ( Fig. 6B,C ). At this stage, the gonads already were differentiated into testis and epididymis ( Fig. 6D ). The testis possessed interstitial cells and the seminiferous tubules ( Figure 6E ) and tubules were present in the epididymis ( Fig. 6F ).

### Comparative aspects of the development of mule conceptuses

Comparison of the morphological structures identified previously in equine conceptuses ( Franciolli et al., 2011 ; Rodrigues et al., 2014 ) at similar stages during gestation indicated that the mule embryonic development is advanced in relation to the horse, in particularly the turning of the embryo (i.e., appearance of C format), presence of branchial archs, limb and tail buds, formation of primary and secondary brain vesicles, heart compartmentalization and visualization of somites.

## Discussion

At 17 days of gestation, the conceptus presented an oval shape, a large yolk sac as well as the primary brain vesicles. The forelimb and tail buds were present earlier than limb buds that were observed at day 25, the same age at which the somites were present. At this age, the optic vesicles were apparent and became pigmented from day 33 onwards, even as the skin became opaque. At 33 days of gestation, the forelimb and hind limb could be clearly identified. The liver and heart were prominent. And the large yolk sac possessed blood vessels that allowed forming a trilaminar omphalopleura. The chorioallantoic membrane was small, but well vascularised. The secondary brain vesicles and spinal cord became evident at 35 days of gestation, as well as the ventricular and subventricular zones differentiated in the lateral ventricles, and the choroid plexus was observed at the fourth ventricle. At day 40, the yolk sac became smaller whereas the chorioallantoic membrane expanded. The choroid plexus epithelium became more cuboidal, as well as the neuro-epithelium around the spinal cord. The heart had the typical format of right and left atria and ventricles separated by an intermediary septum. The lung possessed the bifurcation into the primary bronchi. In the kidney, glomerulus was surrounded by the mesonephric tubules. At day 45, the external ears, neck and nostrils were observed. The telencephalon and diencephalon were present, and the three flexures were found. In the fetus of 51 days, the yolk sac was more regressed. At 63 days, the yolk sac was found only around the umbilical cord, the chorioallantoic membrane was expanded, and the amnion enlarged. The lips, eyelids, hooves and genital tubercles occurred, even as the lissenphalic cerebral hemispheres presented a prominent olfactory bulb. The thalamus occurred in the diencephalon, and the cerebellum occurred dorsal to bridge and the conoid medulla oblongata. The walls of the ventricle were complexly differentiated, the cerebellum had primary fissures and the pons was developed. In the heart, the atria had developed valves and a well-structured myocardium was present in the ventricles. The lung was lobulated and had both primary and second bronchi. The metanephros had the shape of the kidney with glomeruli, capsule, proximal and distal convulated tubules. And the gonads already were differentiated into testis and epididymis.

Crossbreeding is a method to develop more productive animals by obtaining an additive merit for specific characters through the interactions between maternal and paternal alleles that promote genome-wide changes, resulting in hybrid vigor ( Chen, 2010b ). This phenomenon as observed in mules is in agreement with the evidences observed in the differences in fetal and placental weight between inbreed and outbreed rats ( Matthews, Peel, 1991 ), as well as in mouse F1 embryos ( Hetherington, 1973 ; Blakley, 1978 ). The variation in the degree of hybrid vigor during different stages of growth and development also was reported in plants ( Lippman et al., 2008 ).

In beef cattle, economical importance of the cumulative effects of hybrid vigor on individual and maternal traits have been obtained from breed crosses ( Gregory and Cundiff, 1980 ; Long, 1980 ; Kress et al., 1995 ; Brandt et al., 2010 ), resulting in increased production levels ( Cundiff et al., 1992 ; Williams et al., 2010 ; Retallick et al., 2013 ). In sheep, crossbreeding has been used to develop more productive sheep and the magnitude of individual hybrid vigor for litter size and its components ( Land et al., 1974 ; Bradfort et al., 1989 ; Di et al., 2012 ) in which the typical estimates for average lamb weight per ewe varied from 15 to 20% ( Shrestha et al., 1983 ) and for survival from 9 to 15% ( Ferreira et al., 2015 ).

Despite the economic importance and since *equidae* crossbreeding studies have been conducted only in adult mules ( Travis, 1990 ; Proops et al., 2009 ), little is known about the occurrence of hybrid vigor during intrauterine development. Compared to developmental pattern of horse conceptuses, we founded that some structures occurred slightly earlier in the mule. However, besides these advanced developmental features, there were no major differences in most fine anatomical structures between the mule and the equine conceptuses.

More specifically, some structures showed histological similarities between mule and equine conceptuses of the same age, even though they occurred earlier in the mule. For instance, the typical form of the heart and main differentiation of two atrium, two ventricles and the prominent interventricular groove developed very similarly within a period of 30 to 45 days in both mule and equine ( Tab. 2 ; Cottrill et al., 1997 ; Betteridge, 2000 ; Giussani and Fowden, 2005 ; Franciolli et al., 2011 ; Rodrigues et al., 2014 ).

**Table 2:** Comparison of timing of appearance of morphological characteristics inequine and mule conceptuses. The data indicate that most of the structures appear first in the mule conceptuses.

Characteristics			Days of gestation	
			Equine	Mule
Translucient skin			-	17
Format in letter C			28	25
Branchial archs			22	17
Optic vesicles			25	25
Optic cups			21-26	-
Limb buds			25/22	17
Tail			25/28	17
Mesonephro			19 - 25	17
Metanephro			36 - 38	-
Primary brain vesicles	Prosencephalon, Mesencephalon, Rhombencephalon		34	17 to 32
		Optic bulb	50	40
		Cephalic, Pontine and Cervical flexures	40	40
Secondary brain vesicles			Starting from 40	33
	Telencephalon (Tel) Dyencephalon (Dye) Mesencephalon (Mes)	Cerebral hemispheres (CH) Thalamus (Tha) Aqueduct (Aq)	40 – Tel/Dye/Mes 50 – CH/Tha/Aq	33 – Tel/Dye/Mes 63 – CH/Tha/Aq
	Metencephalon	IV Ventricle	40	40
		Pons	50	40
		Cerebellum	50	63
	Myelencephalon	Medulla oblongata	50	40
Retina pigmented			38/32	33
Eyelid formation			38/45	45 - complete
Nostrils formation			38	45
Heart	2 chambers		21	17
	Compartmentalized		28	25
	Definitive position		30 – 45	25 – 45
Liver	Projection abdominal cavity		25	17
Somite			25	17
Spinal cord			-	33
Vertebral bodies			38	45

Likewise, the internal differentiation of lobulation and primary and secondary bronchi inside the lung as well as the inner structure of the meso- and metanephros followed similar principles ( Tab. 2 ; Moore and Persuad, 2004; Franciolli et al., 2011 ). In addition, the differentiation and regionalization of the central nervous system resembled equivalent internal features ( Tab. 2 ; Franciolli et al., 2011 ; Rigoglio et al., 2017 ). Finally, there were also structures that occurred somewhat earlier in the horse compared to the mule, e.g. the cerebral hemispheres, thalamus, aqueduct, and cerebellum inside the central nervous system ( Tab. 2 ; Franciolli et al., 2011 ; Rigoglio et al., 2017 ). Even though we found some temporal adjustments in the occurrences of structures between the mule and the equine, they showed small dissimilarity at internal differentiation, suggesting that hybrid vigor in the intrauterine development is present in the mule, at least with regard to its maternal equine developmental patterns.

According to the “parental conflict of interest hypothesis” (Moor and Haig, 1991), genomic imprinting’s evolutionary explanation is that the father’s genes enhance fetal growth to improve the success of the paternal genome to be passed on. In the case of mules, growth-related paternally-expressed donkey genes such as IGF2 may be upregulated or the growth-restrictive maternally-expressed horse genes such as IGF2R may be downregulated in the mule conceptus, leading to accelerated developmental rates. Further studies to characterize the development characterists of the hinny conceptus (reciprocal cross of a stallion with a jenny) could elucidate whether genomic imprinting plays a role in these divergent developmental rates during gestation. Moreover, horses have shorter gestation intervals (11 months) compared to donkeys (12 months).

Although little is known of the length of mule gestations, a report has shown a 7 day increase in gestation length in mares carrying mule foals ( Giger et al, 1997 ), indicating that mule conceptuses have a longer period to develop and therefore no physiological need to accelerate their developmental rate to meet maternal gestational length. Analysis of the donkey conceptus at similar stages would be necessary to confirm whether the mule development is advanced in comparisson to both parental species. Together, these data show that the mule development at the embryo-to-fetus transition period is accelerated in some features, indicating that either hybrid vigor and/or genomic imprinting may play a role in these developmental divergencies.
